# The Human Oocyte Preservation Experience (HOPE) Registry: evaluation of cryopreservation techniques and oocyte source on outcomes

**DOI:** 10.1186/s12958-017-0228-7

**Published:** 2017-02-07

**Authors:** Zsolt Peter Nagy, Robert E. Anderson, Eve C. Feinberg, Brooke Hayward, Mary C. Mahony

**Affiliations:** 1 Reproductive Biology Associates, 1100 Johnson Ferry Rd #200, Atlanta, GA 30342 USA; 2 Southern California Center for Reproductive Medicine, 361 Hospital Rd #333, Newport Beach, CA 92663 USA; 3 Fertility Centers of Illinois, 67 Park Ave W #190, Highland Park, IL 60035 USA; 4 EMD Serono, Inc., One Technology Pl., Rockland, MA 02370 USA

**Keywords:** Assisted reproductive technology, Slow-freezing, Vitrification, Donor oocytes, Autologous oocytes

## Abstract

**Background:**

This prospective, Phase IV, multicenter, observational registry of assisted reproductive technology clinics in the USA studied outcomes of first cycles using thawed/warmed cryopreserved (by slow-freezing/vitrification) oocytes (autologous or donor).

**Methods:**

Patients were followed up through implantation, clinical pregnancy, and birth outcomes. The main outcome measure was live birth rate (LBR), defined as the ratio of live births to oocytes thawed/warmed minus the number of embryos cryopreserved for each cycle, averaged over all thawing cycles. Clinical pregnancy rate (CPR) was also evaluated, and was defined as the presence of a fetal sac with heart activity, as detected by ultrasound scan performed on Day 35–42 after embryo transfer.

**Results:**

A total of 16 centers enrolled 204 patients; data from 193 patients were available for analyses. For donor oocytes, in the slow-freezing (*n* = 40) versus vitrification (*n* = 94) groups, respectively, CPR and LBR were significantly different: 32.4% versus 62.6%, and 25.0% versus 52.1%; outcomes from Day 3 transfers did not differ significantly. For vitrified oocytes, in the autologous (*n* = 46) versus donor (*n* = 94) group, respectively, CPR and LBR were significantly different: 30.0% versus 62.6% and 17.4% versus 52.1%. This was largely due to a significant difference in CPR with Day 5/6 transfers.

**Conclusions:**

In two subgroup data analyses, in women who received cryopreserved oocytes from donors, CPR and LBR were significantly higher in cycles using oocytes cryopreserved via vitrification versus slow-freezing, reflecting differences in methodologies and more Day 5/6 transfers; in women who received vitrified oocytes, CPR and LBR were significantly higher in cycles using donor versus autologous oocytes with Day 5/6 transfers.

**Trial registration:**

ClinicalTrials.gov: NCT00699400. Registered June 13, 2008.

## Background

Oocyte cryopreservation is a medically recognized treatment option for women at risk of losing their fertility potential, including those needing to undergo cancer therapy and those with premature ovarian insufficiency [1, 2]. It is also increasingly being used by women who, for the purpose of education, health, career, or other reasons, desire to postpone childbearing [3, 4]. Oocyte cryopreservation can be used in *in vitro* fertilization (IVF) as an alternative to cryopreserved embryos, either for legal or ethical reasons [5, 6]. For example, in some countries, such as Germany, it is illegal to freeze embryos. Additionally, cryopreservation of donor oocytes through ‘oocyte banking’ may be a more practical alternative to fresh oocyte donation [7, 8].

The first pregnancy was achieved from a cryopreserved oocyte using the slow-freezing method in 1986 [9]. For nearly two decades, slow-freezing was the primary method of oocyte cryopreservation, yet it was not used routinely in clinical practice due to low efficiency. Vitrification was first reported as an alternative technique for oocyte cryopreservation in humans in 1999 [10], but became more widely used after 2005 when a more efficient protocol was developed by Kuwayama and colleagues [11]. By 2009, more than 50% of assisted reproductive technology (ART) clinics in the US offered oocyte cryopreservation [12] for both fertility preservation and donor oocyte banking.

The Human Oocyte Preservation Experience (HOPE) Registry (ClinicalTrials.gov: NCT00699400) was established to collect information on outcomes from ART cycles that used cryopreserved oocytes and to compare outcomes from two techniques – slow-freezing and vitrification [13–15]. Both techniques involve the cryopreservation of oocytes when they are mature and become available for collection upon oocyte retrieval. In the registry, cryopreserved oocytes could be autologous (patient’s own oocytes) or heterologous (from a donor).

At the time the HOPE Registry was set up in 2008, cryopreservation of oocytes was not widely performed and the Practice Committees of the American Society of Reproductive Medicine (ASRM) and the Society for Assisted Reproductive Technology (SART) considered oocyte cryopreservation to be an experimental technique that required further safety and efficacy evaluation [16]. More recent studies have demonstrated that oocyte cryopreservation, and especially vitrification, does not have a negative impact on oocyte physiology or on the chromosomal status of the embryos derived [17–20]. As a result, in 2013, the Practice Committees of the ASRM and SART reported that, with appropriate patient counseling, oocyte cryopreservation should no longer be considered experimental for patients facing infertility due to chemotherapy or other gonadotoxic therapies [21]. However, the committees considered there were insufficient data to recommend oocyte cryopreservation for the sole purpose of circumventing reproductive aging in healthy women and that more data were needed before this technology should be used routinely in lieu of embryo cryopreservation. In addition, they considered that more widespread clinic-specific data on the safety and efficacy of oocyte cryopreservation in donor populations are needed before universal donor oocyte banking can be recommended. At the same time, the European Society of Human Reproduction and Embryology (ESHRE) has embraced the idea of oocyte cryopreservation for age-related fertility loss [22].

Registries are a valuable complement to randomized controlled trials (RCTs) in determining real-world evidence in all areas of medicine. In contrast to RCTs, they do not generally have restrictive inclusion or exclusion criteria and thus include a broader spectrum of patients. In fact, in this study, heterogeneity was found to be a confounder due to use of both autologous and heterologous oocytes. Therefore, this paper focuses on the subgroup analysis of patients using autologous and donor oocytes and between patients using slow-freezing and vitrification from the HOPE Registry. Since the time the registry was started, vitrification has become a more widely used technique than slow-freezing and this is reflected in the numbers of patients in the two groups.

## Methods

### Ethics, consent and permissions

The HOPE Registry was a prospective, Phase IV, multicenter, observational registry. The study protocol and all major amendments were approved by all relevant Institutional Review Boards, Independent Ethics Committees, and Health Authorities. The registry was conducted in accordance with the International Conference on Harmonization guidelines for Good Clinical Practice, applicable local regulations, and the Declaration of Helsinki.

Patients and partners (if applicable) provided written informed consent. A second informed consent form was completed by patients and partners (if applicable) after a live birth for follow-up data to be collected on babies. The first patient was enrolled to the registry in June 2008. Originally, it was planned that the registry would run for 5 years (3 years for enrollment and 2 years to complete a 12-month well-baby follow-up for all live births). Enrollment was open for 2 of the intended 3 years and closed early on September 15, 2010. Patients already enrolled in the registry continued to be followed to collect data on birth outcomes and child development at 12 months of age. The registry was closed on May 17, 2012.

### Patients

The registry was intended for use by all ART centers in the US that offered oocyte cryopreservation. Patients and donors were women of reproductive age (18–50 years). Patients were undergoing IVF using frozen–thawed or vitrified–warmed oocytes (either their own or from oocyte donors). Patients were excluded if they had clinically significant systemic disease (including cancer), abnormal undiagnosed gynecological bleeding, or any contraindication to controlled ovarian stimulation (COS) or to gonadotropins to be used in ART; or if they were undergoing embryo transfer with mixed embryos generated from fresh oocytes from the current cycle and frozen embryos generated from non-frozen oocytes obtained in a previous ART cycle.

### Methods

Donors and patients underwent COS to retrieve and cryopreserve oocytes. All oocytes that survived the thawing/warming procedures underwent ICSI. Fertilized oocytes were cultured according to clinic standard operating procedures and then transferred to patients. Supernumerary embryos of adequate quality were cryopreserved. Information on the embryo quality and development, as well as transfer procedure, were collected prospectively through electronic clinical report forms. Oocyte cryopreservation could have taken place before, at the time of, or following the registry’s launch, with all thawing/warming, fertilization, and transfer procedures occurring after the patient was enrolled in the registry. Thus, outcomes of cycles using cryopreserved oocytes that were thawed/warmed, fertilized, and transferred to patients were gathered prospectively through electronic clinical report forms. Outcomes from cryopreserved supernumerary embryos were not included in analyses.

Patients were followed up through implantation, clinical pregnancy, and birth outcomes; babies were assessed at birth and followed up at 12 months of age. Regular monitoring across all centers ensured clean patient data. Six centers were audited. The sponsor or representative periodically reviewed electronic case report forms and other Registry documents and conducted verification of source data (all data in original records and copies of original records of clinical findings, observations, or other activities).

### Objectives

The primary objective of the registry was to record prospectively and track the outcomes of cycles using cryopreserved oocytes (autologous or donor) that were thawed/warmed, fertilized, and transferred to patients. The secondary objectives included identifying factors associated with successful cycle outcomes utilizing standard medical practices, such as oocyte survival rate, the number of clinical pregnancies, and implantation rate.

### Subgroup analyses

As patients can choose to use donor oocytes (usually from younger female donors) to overcome age-related infertility problems, the HOPE Registry included the use of both autologous and donor oocytes for cryopreservation. Because there were differences in the mean age of oocytes cryopreserved in the autologous versus donor oocyte groups and in the slow-freezing versus vitrification groups, additional analyses were conducted in two subgroups to extract more homogenous results from the collected data: donor oocyte subgroup by cryopreservation technique used, and vitrified oocytes by donor or autologous subgroup.

### Endpoint analyses

Planned endpoints included: oocyte survival rate (percentage of oocytes thawed/warmed that underwent intracytoplasmic sperm injection [ICSI]), and implantation rate (percentage of embryos transferred that developed fetal sacs with heartbeat). Other endpoints included: number of oocytes thawed/warmed, number of ICSI oocytes, number of two-pronuclear (2PN) oocytes, embryo transfers, number of embryos transferred, embryo cryopreservations, biochemical pregnancy rate (positive beta-human chorionic gonadotropin test), clinical pregnancy rate (the presence of a fetal sac with heart activity, as detected by ultrasound scan performed on Day 35–42 after embryo transfer), and miscarriage rate.

Birth outcomes included: number of live birth deliveries per cycle started, oocyte efficiency (percentage of oocytes thawed/warmed resulting in a live birth), number of births/delivery, number of pre-term babies, and complications with regard to pregnancy, delivery, and perinatal conditions.

Child health was assessed at birth and 12 months and included recording the presence of a congenital abnormality, birth defect, or major anomaly.

Endpoints identified for inclusion in the subgroup analyses included the number of oocytes needed for a pregnancy (defined as the number of oocytes thawed/warmed divided by the number of clinical pregnancies), the number of oocytes needed for a live birth (defined as the number of oocytes thawed/warmed divided by the number of live births), and oocyte efficiency (defined as percentage of oocytes thawed/warmed resulting in a live birth).

### Statistical analyses

The analyses included only data from the first cycle in the registry for each patient. *p* values for comparison between groups were generated from *t*-tests (comparison of means) and from Fisher’s exact tests (comparisons of percentages). A *p* value of < 0.05 was considered to denote a significant difference. Descriptive statistics are also presented by age group (oocyte age <35 and ≥35 years) for patients receiving autologous oocytes in the vitrification group for comparison with the younger donor oocyte ages.

The number of patients in the HOPE Registry determined the sample size for the subgroup analyses.

## Results

### Patient disposition (all patients)

**Fig. 1:**
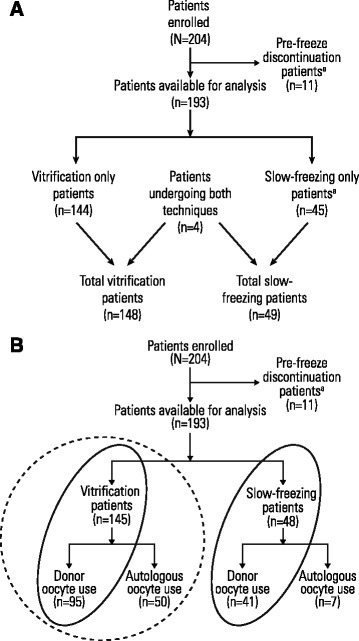
Patient disposition: **a** all patients enrolled in Registry; **b** donor oocyte and vitrification subgroup analyses. Patients included in the donor oocyte subgroup analysis are circled with a solid line and those included in the vitrification subgroup analysis are circled with a dashed line. ^a^ Pre-freeze discontinuation patients denotes enrolled patients who were withdrawn before oocyte retrieval or for whom oocyte retrieval failed (ie, patients who had no oocytes to freeze)

A total of 16 centers enrolled 204 patients, and data from 193 patients were available for analyses; 11 patients discontinued before oocyte cryopreservation due to either failed ovarian stimulation or patient withdrawal (Fig. 1).

In total, 206 cryopreservation cycles and 209 thawing/warming cycles were performed for these 193 patients: 45 patients underwent ≥1 slow-freezing cycle only; 144 patients underwent ≥1 cycle using vitrification only; 4 patients underwent cycles using both techniques. All but two patients, both of whom had undergone a single vitrification cycle, subsequently underwent ≥1 cycle of oocyte thawing/warming. Most patients underwent a single cycle of oocyte cryopreservation and a single cycle of oocyte thawing/warming (173/193, 89.6%; 40 and 133 in the slow-freezing and vitrification groups, respectively); 5/193 (2.6%) underwent a second thawing/warming from the first freeze (two in the slow-freezing group and three in the vitrification group); 9/193 (4.7%) underwent a second freeze and second thawing/warming using the same technique (three in slow-freezing group and six in the vitrification group). Of the four patients who switched to a different cryopreservation method for their second cycle, three in the slow-freezing group switched to the vitrification group and one in the vitrification group switched to the slow-freezing group. They each thawed/warmed once after each cryopreservation.

### Patient characteristics

In subgroup analyses, no difference was observed in the age of women receiving donor oocytes in the slow-freezing versus vitrification groups (*p* = 0.95; Table 1). However, there was a trend for donor oocytes to be younger in the slow-freezing versus vitrification groups (*p* = 0.08; Table 1). There was also a trend for women using their own oocytes to be older in the slow-freezing versus vitrification groups: 36.9 ± 1.8 versus 33.9 ± 3.9 years, respectively (*p* = 0.05; Table 1). The indications for oocyte cryopreservation for patients using autologous oocytes were as follows: vitrification: 6/50 (12%) medical, 42/50 (84%) proof of concept, and 36/50 (72%) elective; slow-freezing: 5/7 (71%) medical, 4/7 (57%) proof of concept, and 7/7 (100%) elective. More than one indication for oocyte cryopreservation could be reported.

**Table 1:** Demographic/clinical characteristics of patients enrolled in the donor and autologous subgroups of the HOPE Registry

Age at earliest cryopreservation (years)	Slow-freezing	Vitrification	*p* value^a^
Patients using donor oocytes	*n* = 41	*n* = 95	
Patient age, mean ± SD; range	40.2 ± 5.2; 23–47	40.2 ± 4.7; 24–51	0.9526
Oocyte age, mean ± SD; range	25.1 ± 2.7; 20–31	26.0 ± 2.8; 21–31	0.0835
Patients using autologous oocytes	*n* = 7	*n* = 50	
Patient and oocyte age, mean ± SD; range	36.9 ± 1.8; 35–40	33.9 ± 3.9; 25–43	0.0528

*HOPE* Human Oocyte Preservation Experience; *IVF in vitro* fertilization; *SD* standard deviation

^a^ *p* value from *t*-test for continuous data

### Subgroup analyses

#### Donor oocyte group: slow-freezing versus vitrification

The analysis of donor oocytes included outcomes from the first thawing/warming cycles of 136 patients: 41 in the slow-freezing group and 95 in the vitrification group (Fig. 1a). Mean oocyte age and mean number of oocytes thawed/warmed per cycle did not differ significantly between groups (Table 2).

**Table 2:** Oocyte characteristics and ART outcomes in women receiving donor oocytes frozen by slow-freezing or vitrification

	Slow-freezing (*n* = 41)	Vitrification (*n* = 95)	*p* value^a^
Oocyte age (years), mean ± SD; range	25.1 ± 2.7; 20–31	26.0 ± 2.8; 21–31	0.08
Total number of cycles started	40	94	
Total number of oocytes warmed/thawed	312	694	
Oocytes warmed/thawed per cycle, mean ± SD	7.8 ± 2.8	7.4 ± 2.5	0.40
Oocyte survival rate,^b^ mean ± SD	72.3% ± 28.3%	86.0% ± 19.4%	< 0.01
2PN oocytes, mean ± SD	4.0 ± 1.7	5.2 ± 1.8	< 0.01
Embryo transfers,^c^ n (% of cycles)	37 (92.5)	91 (96.8)	0.36
Transfers on Day 3,^d^ n (% of transfers)	31 (83.8)	19 (20.9)	< 0.01
Transfers on Day 5/6,^d^ n (% of transfers)	0 (0.0)	68 (74.7)	
Transfers on unknown/other days,^d^ n (% of transfers)	6 (16.2)	4 (4.4)	
Embryos transferred per cycle, mean ± SD	2.9 ± 1.0	2.1 ± 0.6	< 0.01
Day 3 transfer,^d^ mean ± SD	2.7 ± 1.0	2.7 ± 0.7	0.99
Day 5/6 transfer,^d^ mean ± SD	0	2.0 ± 0.4	< 0.01
Transfer on unknown/other days,^d^ mean ± SD	3.5 ± 0.8	2.3 ± 0.5	0.03
Embryo cryopreservations, n (% of cycles)	4 (10.0)	50 (53.2)	< 0.01
Embryos cryopreserved, mean ± SD	1.8 ± 0.5	2.3 ± 1.1	0.34
Implantation rate,^e^ mean ± SD	14.4% ± 23.9%	50.4% ± 45.8%	< 0.01
Day 3 transfer, mean ± SD	16.3% ± 25.4%	12.3% ± 22.1%	0.57
Day 5/6 transfer, mean ± SD	0	61.0% ± 45.7%	Not done
Transfer on unknown/other days,^d^ mean ± SD	4.2% ± 10.2%	50.0% ± 40.8%	0.19
Clinical pregnancies, n (% of transfers)	12 (32.4)	57 (62.6)	< 0.01
Day 3 transfer,^d^ n (% of transfers)	11 (35.5)	5 (26.3)	0.72
Day 5/6 transfer,^d^ n (% of transfers)	0	49 (72.1)	Not done
Transfer on unknown/other days,^d^ n (% of transfers)	1 (16.7)	3 (75.0)	0.24
Number of oocytes needed for a pregnancy^f^	17.3	7.5	< 0.01
Live birth deliveries, n (% of cycles)^g^	10 (25.0)	49 (52.1)	< 0.01
Number of oocytes needed for a live birth^h^	22.3	9.3	< 0.01
Oocyte efficiency, number of live births (% of warmed/thawed oocytes)^i^	14 (4.5)	75 (10.8)	< 0.01

*2PN* two-pronuclear; *ART* assisted reproductive technology; *ICSI* intracytoplasmic sperm injection; *SD* standard deviation

^a^ *p* values for comparison between groups are from *t*-tests for comparison of means and from Fisher’s exact tests for comparisons of percentages

^b^ Percentage of oocytes warmed that underwent ICSI

^c^ Patients not undergoing embryo transfer had no oocytes to transfer (ie, they failed at a stage between oocyte warm/thaw and transfer)

^d^ Embryo transfer date minus oocyte warming date

^e^ Percentage of embryos transferred that successfully undergo implantation and develop fetal sacs with heartbeat

^f^ Number of oocytes warmed/thawed divided by number of fetal sacs with heartbeat

^g^ Percentage of cycles started resulting in live birth of one or more babies

^h^ Number of oocytes warmed/thawed divided by number of live births

^i^ Percentage of oocytes warmed resulting in a live birth

The mean oocyte survival rate and the mean number of oocytes fertilized (2PN oocytes) were significantly higher in the vitrification group than in the slow-freezing group (Table 2). Embryo transfers in the slow-freezing group primarily occurred on Day 3 (84% per cycle started; none on Day 5/6), while most in the vitrification group occurred at blastocyst stage (75% on Day 5/6; 21% on Day 3). The mean number of embryos transferred per cycle was significantly lower in the vitrification group than in the slow-freezing group (Table 2). For embryos transferred on Day 3, there was no significant difference between the mean number of embryos transferred in the slow-freezing and vitrification groups.

In cycles from which embryo cryopreservation was performed, the mean ± SD number of embryos cryopreserved was similar in the two groups (1.8 ± 0.5 and 2.3 ± 1.1; *p* = 0.34). Totals of 7 and 114 embryos were cryopreserved at rates of 0.18 and 1.21 embryos per cycle started in the slow-freezing and vitrification groups, respectively. The number of embryos cryopreserved as a percentage of oocytes thawed/warmed was significantly lower with slow-freezing (7/312, 2.2%) than with vitrification (114/694, 16.4%; *p* < 0.01); this was also true for embryo cryopreservations on Day 5/6 (2/312 [0.6%] vs 108/694 [15.6%], *p* = 0.02), but a greater percentage of embryo cryopreservations per transfer occurred in the slow-freezing group versus the vitrification group on Day 3 (5/312 [1.6%] vs 1/694 [0.1%], *p* < 0.01).

Implantation and clinical pregnancy rates were significantly higher in the vitrification group than in the slow-freezing group (Table 2). In the slow-freezing group, all implantations and pregnancies were from embryos transferred on Day 3, whereas for vitrification, the majority of implantations and clinical pregnancies were from embryos transferred on Day 5/6. The miscarriage rate (% of biochemical or clinical pregnancies) was not significantly different between groups: 2/14 (14.3%) in the slow-freezing group and 8/65 (12.3%) in the vitrification group. Overall oocyte efficiency (live births per oocyte warmed) was significantly higher in the vitrification group than in the slow-freezing group (Table 2).

The number of singleton live births (% of deliveries) was 6/10 (60%) in the slow-freezing group and 23/49 (46.9%) in the vitrification group. The number of twin births (% of deliveries) was 4/10 (40%) and 26/49 (53.1%), respectively (*p* = not significant). There were no pre-term deliveries in the slow-freezing group and 4 singleton pre-term births and 21 twin pre-term births in the vitrification group. In the slow-freezing group, mean ± SD birth weight of singleton births at term was 3543.7 g ± 370.7 (*n* = 3; *n* missing = 2; one not reported as term or pre-term) and of twin births at term was 2556.2 g ± 566.3 (*n* = 6; *n* missing = 2). In the vitrification group, mean ± SD birth weight of singleton births at term was 3638.2 g ± 305.0 (*n* = 9, *n* missing = 10) and of twin births at term was 2083.7 g ± 188.7 (*n* = 6; *n* missing = 4).

There were no congenital anomalies in the slow-freezing group. In the vitrification group, there were two non-major anomalies (both male; both using donor oocytes): small right split thumb, removed at 4 months; and mild ventricular septal defect and moderate patent foramen ovale, which self-corrected within 12 months. There were no major anomalies in either group. At 12 months, for babies with data available, developmental milestones had been achieved by all five children in the slow-freezing group (four missing follow-up) and in 27/28 (96.4%) in the vitrification group.

#### Vitrified oocyte group: donor versus autologous oocyte use

The analysis of vitrified oocytes included warming cycles from 145 patients: 50 in the autologous group and 95 in the donor group (Fig. 1b). Patient/donor age at the time of oocyte cryopreservation (oocyte age) was significantly lower in the donor oocyte group than in the autologous oocyte group (Table 3).

**Table 3:** Oocyte characteristics and ART outcomes in women receiving autologous versus donor vitrified oocytes

	Autologous oocyte use			Donor oocyte use (*n* = 95)	*p* value^a^
	Oocyte age <35 years (*n* = 24)	Oocyte age ≥35 years (*n* = 26)	All ages (*n* = 50)		
Oocyte age (years), mean ± SD; range	30.5 ± 2.2; 25–34	37.3 ± 1.9; 35–43	33.9 ± 3.9; 25–43	26.0 ± 2.8; 21–31	< 0.01
Total number of cycles started	21	25	46	94	
Total number of oocytes warmed	194	231	425	694	
Oocytes warmed per cycle, mean ± SD	9.2 ± 4.1	9.2 ± 4.1	9.2 ± 4.1	7.4 ± 2.5	< 0.01
Oocyte survival rate,^b^ mean ± SD	70.9% ± 21.4%	84.2% ± 20.5%	78.1% ± 21.7%	86.0% ± 19.4%	0.03
2PN oocytes, mean ± SD	5.1 ± 3.2	5.4 ± 3.7	5.2 ± 3.5	5.2 ± 1.8	0.99
Embryo transfers,^c^ n (% of cycles)	19 (90.5)	21 (84.0)	40 (87.0)	91 (96.8)	0.06
Transfers on Day 3,^d^ n (% of transfers)	10 (52.6)	12 (57.1)	22 (55.0)	19 (20.9)	< 0.01
Transfers on Day 5/6,^d^ n (% of transfers)	8 (42.1)	9 (42.9)	17 (42.5)	68 (74.7)	< 0.01
Embryos transferred per cycle, mean ± SD	2.4 ± 0.9	3.0 ± 1.0	2.7 ± 1.0	2.1 ± 0.6	< 0.01
Day 3 transfer,^d^ mean ± SD	2.8 ± 1.0	3.3 ± 1.1	3.0 ± 1.1	2.7 ± 0.7	0.30
Day 5/6 transfer,^d^ mean ± SD	1.9 ± 0.4	2.6 ± 0.7	2.2 ± 0.7	2.0 ± 0.4	0.13
Embryo cryopreservations, n (% of cycles)	4 (19.0)	6 (24.0)	10 (21.7)	50 (53.2)	< 0.01
Embryos cryopreserved, mean ± SD	1.8 ± 1.0	3.5 ± 1.8	2.8 ± 1.7	2.3 ± 1.1	0.37
Implantation rate,^e^ mean ± SD	19.3% ± 33.5%	11.5% ± 19.5%	15.2% ± 26.9%	50.4% ± 45.8%	< 0.01
Day 3 transfers, mean ± SD	11.7% ± 19.3%	11.8% ± 18.3%	11.7% ± 18.3%	12.3% ± 22.1%	0.92
Day 5/6 transfers, mean ± SD	31.3% ± 45.8%	11.1% ± 22.0%	20.6% ± 35.6%	61.0% ± 45.7%	< 0.01
Clinical pregnancies, n (% of transfers)	6 (31.6)	6 (28.6)	12 (30.0)	57 (62.6)	< 0.01
Day 3 transfers,^d^ n (% of transfers)	3 (30.0)	4 (33.3)	7 (31.8)	5 (26.3)	0.74
Day 5/6 transfers,^d^ n (% of transfers)	3 (37.5)	2 (22.2)	5 (29.4)	49 (72.1)	< 0.01
Live birth deliveries, n (% of cycles)^f^	5 (23.8)	3 (12.0)	8 (17.4)	49 (52.1)	< 0.01
Number of oocytes needed for a live birth^g^	38.8	77.0	53.1	9.3	< 0.01
Oocyte efficiency, number of live births (% of warmed/thawed oocytes)^h^	5 (2.6)	3 (1.3)	8 (1.9)	75 (10.8)	< 0.01

*2PN* two-pronuclear; *ART* assisted reproductive technology; *ICSI* intracytoplasmic sperm injection; *SD* standard deviation

^a^ *p* values for comparison between patients using autologous oocytes (all ages) versus patients using donor oocytes are from *t*-tests for comparison of means and from Fisher’s exact tests for comparisons of percentages

^b^ Percentage of oocytes warmed that underwent ICSI

^c^ Patients not undergoing embryo transfer had no oocytes to transfer (ie, they failed at a stage between oocyte warm/thaw and transfer)

^d^ Embryo transfer date minus oocyte warming date. Remaining embryo transfers for each group were performed on other days

^e^ Percentage of embryos transferred that successfully undergo implantation and develop fetal sacs with heartbeat

^f^ Percentage of cycles started resulting in live birth of one or more babies

^g^ Number of oocytes warmed divided by number of live births

^h^ Percentage of oocytes warmed resulting in a live birth

Significantly fewer oocytes per cycle were warmed in the donor group (Table 3), and oocyte survival rate was higher in the donor group (Table 3). The mean number of fertilized oocytes (2PN oocytes) was not significantly different between groups (Table 3). However, fertilization rate (number of 2PN oocytes per total number of oocytes undergoing ICSI) was significantly higher in the donor group than in the autologous group (84.3% vs 74.3%, respectively). Most embryo transfers in the autologous group occurred on Day 3 (55%; 43% on Day 5/6) and most in the donor group occurred on Day 5/6 (75%; 21% on Day 3). The majority of transfers for both autologous oocyte use age groups (age <35 years and ≥35 years) occurred on Day 3; however, the number of embryos transferred per cycle was lower in patients aged <35 years than in older patients, and in this respect the younger patients were similar to patients using donor oocytes. The mean number of embryos transferred per cycle was significantly lower in the donor oocyte group (Table 3); no significant differences between autologous and donor groups were seen when comparing mean numbers of embryos transferred on Day 3 and on Day 5/6. The total number of embryos cryopreserved was 28 in the autologous group and 114 in the donor group. The number of embryo cryopreservations as a percentage of cycles was significantly lower in the autologous group than in the donor group (*p* < 0.01; Table 3), but there was no significant difference in mean ± SD number of embryos cryopreserved in the autologous versus donor groups (2.8 ± 1.7 vs 2.3 ± 1.1, respectively, *p* = 0.37).

Implantation, clinical pregnancy, and LBR were significantly lower among women receiving autologous oocytes compared with those receiving donor oocytes (Table 3). However, implantation and clinical pregnancy rates were not significantly different for Day 3 embryo transfers, but were significantly different for Day 5/6 transfers. Despite a similar number of Day 5/6 embryos transferred for patients aged <35 years using autologous oocytes, implantation and clinical pregnancy rates remained lower than for patients using donor oocytes. The miscarriage rate (% of biochemical or clinical pregnancies) was not significantly different between groups: 2/23 (8.7%) in the autologous group and 8/65 (12.3%) in the donor group.

The number of singleton live births (% of deliveries) was 8/8 (100%) in the autologous group and 23/49 (46.9%) in the donor group. The number of twin births (% of deliveries) was 0/8 (0%) and 26/49 (53.1%), respectively. One singleton birth was pre-term in the autologous group. In the donor group, five singleton births and 21 twin births were pre-term. There were no congenital anomalies in the autologous group. In the donor group, two non-major anomalies were reported, as detailed above. At 12 months, for babies with data available, developmental milestones had been achieved by all six children in the autologous group and in 27/28 (96.4%) in the donor group.

## Discussion

The ASRM and SART have reported that they would like to see more data on safety, efficacy, ethics, emotional risks, and cost effectiveness from women using cryopreservation to delay childbearing [21]. In this respect, the efficacy data provided by the HOPE Registry may be useful for consideration by the ASRM and SART regarding guidelines on the use of oocyte cryopreservation.

In the time since Registry closure in May 2012, the oocyte cryopreservation technique of vitrification has been used increasingly, even in countries where slow-freezing of oocytes is well established [23]. The HOPE Registry was started when both methods were in use and one of its objectives was to compare the two cryopreservation techniques. The data from 16 clinics represent 3.7% of the IVF clinics in the US (*N* = 436) in 2008, and reflect the small number of clinics offering oocyte cryopreservation (estimated at *N* = 30) at this time [24].

Age of oocyte had a statistically significant effect on outcomes, suggesting that patients compensated for age by using donated oocytes. Heterogeneity was found to be a confounder due to the inclusion of patients who used autologous oocytes and patients who used donor oocytes. Consequently, this paper focuses on subgroup analyses. One subgroup analysis compared outcomes with slow-freezing and vitrification in patients receiving donor oocytes. Another compared outcomes with autologous and donor oocytes that had been vitrified. An RCT performed between 2011 and 2013 in patients receiving autologous oocytes found LBR per embryo thawed/warmed to be significantly higher after vitrification versus slow-freezing when using Day 3 embryos [25].

From the subgroup analysis of data from women who received cryopreserved oocytes from donors, even though the mean number of oocytes thawed/warmed per cycle were similar, some outcomes – including the mean number of 2PN oocytes per cycle, oocyte survival rate, implantation rates, clinical pregnancy rates, and LBRs – were significantly higher in cycles using oocytes cryopreserved via vitrification than in those using oocytes cryopreserved via slow-freezing. Most embryo transfers occurred on Day 3 in the slow-freezing group and on Day 5/6 in the vitrification group. The differences in outcomes observed may be due to a number of reasons: the differences in the cryopreservation technology employed in the two techniques, differences in clinic practices, or the number of embryo transfers on Day 5/6. While the decision on what day to perform embryo transfers – Day 3 (cleavage stage) versus Day 5/6 (blastocyst stage) – may depend on clinical practice, it is also often influenced by the number and the quality of embryos available on Day 3: if the patient has a higher number of good-quality embryos available on Day 3, embryo transfer on Day 5/6 is more likely. There were no differences in outcomes between the slow-freezing and vitrification groups when transfers occurred on Day 3. In keeping with the literature, when Day 5/6 transfers were employed in the donor oocyte vitrification group, outcomes were higher than with Day 3 transfers [26]. Of note, both slow-freezing and vitrification resulted in outcomes not dissimilar to those reported by the Centers for Disease Control and Prevention in 2009 for women using fresh cycles (cycles resulting in pregnancies 36.9%, cycles resulting in live births 30.0%) [27], which is of particular significance in a registry setting where clinical practice varies among clinics.

From the second subgroup analysis of vitrified oocytes from autologous and donor oocyte use, significantly better outcomes – including the oocyte survival rate, clinical pregnancy, and LBRs – were achieved in cycles using donor oocytes than in those using autologous oocytes. As with the other analysis, most embryo transfers occurred on Day 5/6 in the vitrification group.

Oocyte age was significantly lower in the donor oocyte group than in the autologous oocyte group. Oocyte age is highly predictive of IVF outcomes [28]. More oocytes were thawed in the autologous group than in the donor group, presumably because investigators expected differences in survivability and fertilization; this resulted in a similar number of 2PN oocytes in the two groups. The number of embryos transferred on either Day 3 or Day 5 did not differ significantly between the two groups. While no differences in implantation rate and clinical pregnancy rate were observed with Day 3 transfers, large differences were observed when embryos were transferred on Day 5. Although Day 5/6 embryo quality was not determined in this study, it is expected that embryos transferred on Day 5 will have a higher viability than those transferred on Day 3 [29].

Results published by the Italian National ART Registry over the period of 2007 to 2011 have also shown significantly higher oocyte survival rates and pregnancy rates, per started cycle and per transfer, with vitrification than with slow-freezing of oocytes [23]. Outcomes from 14,328 cycles with 11,599 embryo transfers were reported; clinical pregnancy rates per transfer were 18.0% with vitrification and 14.8% with slow-freezing. In accordance with Italian law, between 2007 and 2009 no more than three embryos could be generated per patient, although this restriction was lifted after 2009. Thus, in the Italian Registry, not all the oocytes that survived the cryopreservation/thawing or warming process were used, which possibly influenced pregnancy rates. Clinical pregnancy rates in the overall patient population of the HOPE Registry population are similar to those reported in the Italian Registry, but those in the HOPE subgroup analyses are higher, particularly those in the donor oocyte subgroup. There was no donor oocyte use in the Italian Registry. In contrast to the Italian Registry, the HOPE Registry is a prospective study, although information on some donors was collected retrospectively.

A recent literature review involving 180 studies has shown that vitrified oocytes produce superior IVF results to slow-frozen oocytes and may yield comparable outcomes to IVF with fresh oocytes in certain patient populations [30]. Patients at risk of infertility due to disease or age-related decline, couples who fail to produce semen when required for IVF, and patients who cannot use cryopreserved embryos for legal or ethical reasons may access cryopreserved oocytes; it has been suggested that these patients should be offered vitrified oocytes [29]. The authors suggest that further research is required to confirm IVF success across all patient populations and to determine the best cryopreservation protocols. In support of the use of vitrified oocytes in IVF, an RCT that compared ongoing pregnancy rates (OPRs) from IVF cycles using vitrified oocytes from an ovum donation program with fresh oocytes found no difference in OPRs between the groups [31]. In our study, in the donor oocyte group, two congenital anomalies (thumb defect and mild ventricular septal defect with moderate patent foramen ovale) were reported in the vitrification group, and none were reported in the slow-freezing group; no anomalies were reported in the autologous oocyte group. These data are in keeping with a review of 936 live births from cryopreserved oocytes (from slow-freezing and vitrification) that reported a 1.3% incidence of birth anomalies, which is comparable with the incidence in naturally conceived infants [32].

Strengths of the HOPE Registry include that it was a prospective study providing ‘real-world’ data. Few registries on ART outcomes following oocyte cryopreservation are available, and most report retrospective data. For example, the ESHRE registry on ART outcomes is a retrospective study that collects data from about 35 countries (number varies by year of reporting) with methods of reporting varying by country [33]. The Australian Institute of Health and Welfare (AIHW) ART Registry also reports data collected retrospectively, although the AIHW registry does involve some data validation, this appears to be limited to erroneous data entries. The AIHW registry also follows up pregnancy and birth outcomes, but the data are limited [34]. Several countries, including France, Switzerland, and Germany [35–37], have their own IVF registries, but few compare outcomes by the methods of cryopreservation.

Other strengths of the HOPE Registry are that information was provided on the number of embryos available for cryopreservation, impact of Day 3 and Day 5 transfers, impact of oocyte number, live birth outcomes, and 12-month follow-up of babies. Cumulative pregnancy rates are considered to be an important outcome [38] and will be influenced by the number of embryos cryopreserved for use in later IVF cycles. Some centers participating in the HOPE Registry were audited (representing 61% of the total study population), and regular monitoring across all centers ensured clean patient data.

Limitations of the HOPE Registry include general limitations of registries such as selection bias due to the inclusion of non-sequential patients, data are generally not 100% verified, data are observational, and missing data. Specifically, HOPE Registry limitations include general changes in the use of cryopreservation techniques in the real world following the start of the registry. In addition, baby follow-up data proved difficult to obtain, probably due to the fact that the investigative site was the ART clinic and obstetricians and pediatricians were involved in delivery and care of babies. For future registry studies, it is recommended to involve the obstetricians and pediatricians early in the study.

## Conclusions

Results from the HOPE Registry provide important information on outcomes following oocyte cryopreservation of both donor and autologous oocytes using slow-freezing and vitrification techniques. This real-world study adds to the pool of evidence for oocyte cryopreservation use in US practices.

## Abbreviations

2PN: Two-pronuclear
AIHW: Australian Institute of Health and Welfare
ART: Assisted reproductive technology
ASRM: American Society of Reproductive Medicine
COS: Controlled ovarian stimulation
CPR: Clinical pregnancy rate
ESHRE: European Society of Human Reproduction and Embryology
HOPE: Human Oocyte Preservation Experience
ICSI: Intracytoplasmic sperm injection
IVF: *In vitro* fertilization
LBR: Live birth rate
OPR: Ongoing pregnancy rate
RCT: Randomized controlled trial
SART: Society for Assisted Reproductive Technology
SD: Standard deviation
